# Extracerebral Tissue Damage in the Intraluminal Filament Mouse Model of Middle Cerebral Artery Occlusion

**DOI:** 10.3389/fneur.2017.00085

**Published:** 2017-03-13

**Authors:** Markus Vaas, Ruiqing Ni, Markus Rudin, Anja Kipar, Jan Klohs

**Affiliations:** ^1^Institute for Biomedical Engineering, University of Zurich, ETH, Zurich, Switzerland; ^2^Institute of Pharmacology and Toxicology, University of Zurich, Zurich, Switzerland; ^3^Institute of Veterinary Pathology, University of Zurich, Zurich, Switzerland

**Keywords:** middle cerebral artery occlusion, mouse, ischemia, external carotid artery, muscle degeneration

## Abstract

Middle cerebral artery occlusion is the most common model of focal cerebral ischemia in the mouse. In the surgical procedure, the external carotid artery (ECA) is ligated; however, its effect on the tissue supplied by the vessel has not been described so far. C57BL/6 mice underwent 1 h of transient MCAO (tMCAO) or sham surgery. Multi-spectral optoacoustic tomography was employed at 30 min after surgery to assess oxygenation in the temporal muscles. Microstructural changes were assessed with magnetic resonance imaging and histological examination at 24 h and 48 h after surgery. Ligation of the ECA resulted in decreased oxygenation of the left temporal muscle in most sham-operated and tMCAO animals. Susceptible mice of both groups exhibited increased *T*_2_ relaxation times in the affected muscle with histological evidence of myofibre degeneration, interstitial edema, and neutrophil influx. Ligatures had induced an extensive neutrophil-dominated inflammatory response. ECA ligation leads to distinct hypoxic degenerative changes in the tissue of the ECA territory and to ligature-induced inflammatory processes. An impact on outcome needs to be considered in this stroke model.

## Introduction

Rodent models of cerebral ischemia are employed for studying pathophysiological mechanisms and for preclinical drug testing (1). Among them, proximal middle cerebral artery occlusion (MCAO) with an intraluminal suture is a well-characterized and widely accepted model of focal cerebral ischemia. In MCAO, the common carotid artery (CCA) is surgically exposed, and a suture is inserted into the CCA, advanced into the internal carotid artery (ICA) until the branch of the middle cerebral artery (MCA), to transiently or permanently interrupt blood flow to the MCA territory. The procedure requires ligation of the external carotid artery (ECA) and thereby also restricts perfusion of the extracerebral ECA territory. In rats, hyperintensities in *T*_2_-weighted magnetic resonance images (MRI) and histological evidence of myofibre degeneration and regeneration were reported on day 5 after MCAO (2). ECA ligation was associated with delayed body weight recovery and impaired motor recovery compared to procedures where the ECA was not ligated (2, 3).

Despite the wide usage of the mouse MCAO model, the effects of ECA ligation have not so far been investigated in this species. In a recent study, we observed neutrophil influx into the ipsilateral temporal muscles in mice after 1 h of transient MCAO (tMCAO) or sham surgery (4). This has prompted us to further investigate the effects of ECA ligation on tributary tissues in the mouse MCAO model.

## Materials and Methods

### Animals

All procedures conformed to the national guidelines of the Swiss Federal act on animal protection and were approved by the Cantonal Veterinary Office Zurich (Permit Number: 18-2014). All procedures fulfill the ARRIVE guidelines on reporting animal experiments. Male C57BL/6J mice (Janvier, France), weighing 20–25 g, of 8–10 weeks of age were used. Animals were housed in a temperature controlled room in individually ventilated cages, under a 12-h dark/light cycle. Paper tissue was given as environmental enrichment. Access to pelleted food (3437PXL15, CARGILL) and water were provided *ad libitum*.

### Study Design

Using G*Power 3.1 software (Heinrich-Heine-University, Düsseldorf, Germany; http://www.gpower.hhu.de/), a group size of *n* = 4 was determined *a priori* for the primary end point *T*_2_ relaxation time, with an effect size *d* = 2.0, α = 0.05 and β = 0.2. Consequently, group sizes of *n* > 4 were used.

In total, 45 mice were randomly allocated to tMCAO or sham surgery (Table 1). For randomization, the web tool www.randomizer.org was used. Animals received randomized numbers and were randomly allocated to their cages and groups. Surgeries were performed in a random order, while the experimenter was blinded to the group until the insertion of the filament during the surgery.

**Table 1:** **Overview summarizing the number of animals for each study, with number of animals that were excluded from final analysis**.

Total animals: *n* = 45				
		Total	Excluded	Exclusion criteria
MSOT	Sham	10	3	Non-responder
	tMCAO	9	2	Non-responder
MRI	Sham 24 h	11	6	*n* = 1 non-responder; *n* = 5 technical reasons
	Sham 48 h	11	1	Non-responder
	tMCAO 24 h	7	2	*n* = 1 non-responder; *n* = 1 technical reasons
	tMCAO 48 h	7	1	Non-responder
Histology	Sham	4	0	
	tMCAO	4	0	

The number of animals that were used for each study and number of animals that were excluded from final analysis are depicted in Table 1. One group of animals underwent assessment of muscle tissue oxygenation with multispectral optoacoustic tomography (MSOT) at 30 min after induction of tMCAO or sham surgery. A second group of animals underwent MRI examination at 24 and 48 h after reperfusion. Due to technical reasons, fewer animals were measured at the 24 h than at the 48 h time point. A third group of animals were transcardially perfused and prepared for histological analysis at 24 h after reperfusion. The unaffected contralateral side was considered to reflect the baseline values. We performed evaluation of all read-out parameters, while blinded to the experimental groups.

The following criteria were used to exclude animals from the end-point analysis: (i) subarachnoidal or intracerebral hemorrhage, (ii) no reflow after filament withdrawal, (iii) Bederson score = 0 (only in MCAO groups), and (iv) dead before experimental endpoint.

### Transient MCAO

Surgical procedures were performed as described previously (4, 5). In brief, anesthesia was initiated by using 3% isoflurane (Abbott, Cham, Switzerland) in a mixture of O_2_ (200 ml/min) and air (800 ml/min) and maintained with 1.5–2% isoflurane. Before the surgical procedure, a local analgesic (Lidocaine, 0.5%, 7 mg/kg) was administered subcutaneously. Temperature was controlled during the surgery and kept constant at 36.5 ± 0.5°C with a feedback-heating controlled pad system. For tMCAO, a midline neck incision was made and the left CCA was ligated proximal of the bifurcation of the ICA and ECA. Subsequently, the left ECA was isolated and ligated and a suture was placed around the ICA. A small incision was made in the CCA and an 11-mm long silicone monofilament (701956PK5Re; Doccol Corporation, USA) was introduced and advanced until it occluded the MCA and left in place for 60 min. A suture around the ICA secured the filament in position. Animals were transferred to a heated recovery box and allowed to wake up. Animals were reanaesthesized, the filament was withdrawn and the ICA was ligated. Sham operation involved surgical procedures, without occlusion of the MCA. After surgery, buprenorphine was administered as s.c. injection (Temgesic, 0.1 mg/kg body weight) and animals were placed in a heated recovery box for 2 h. Buprenorphine was then given twice s.c. every 6–8 h on the day of surgery and thereafter supplied *via* drinking water (1 mg/kg) for 36 h. Animals received softened chow in a petri dish placed on the floor of their cages to encourage eating.

### Multi-Spectral Optoacoustic Tomography

For MSOT, mice were anesthetized with 1.5–2% isoflurane in an oxygen/air mixture (1:4). The fur overlying the head was first trimmed with an electrical shaver and then hair was removed by using a depilation cream (Nair). Mice were placed in a mouse holder in supine position. Isoflurane anesthesia (1.5%) in an oxygen/air mixture was supplied *via* a nose cone. Ultrasound gel (Diagramma, Switzerland) was applied to the mouse head for ultrasonic coupling and the animals were wrapped in a polyethylene membrane. The mouse holder was placed in an acquisition chamber filled with water for acoustic coupling. Water was kept 34°C to maintain body temperature while imaging.

The MSOT inVision 128 small animal imaging system was previously described (6). Briefly, a tunable optical parametric oscillator pumped by an Nd:YAG laser provides excitation pulses with a duration of 9 ns at wavelengths from 680 to 980 nm at a repetition rate of 10 Hz with a wavelength tuning speed of 10 ms and a peak pulse energy of 100 mJ at 730 nm. Ten arms of a fiber bundle provide even illumination of a ring-shaped light strip of approximately 8 mm width. For ultrasound detection, 128 cylindrically focused ultrasound transducers with a center frequency of 5 MHz (60% bandwidth), organized in a concave array of 270° angular coverage and a radius of curvature of 4 cm, were used. Imaging was performed at six wavelengths (715, 730, 760, 800, 850 to 900 nm), at 35–40 consecutive slices with a step size of 0.3 mm, and at 10 averages.

### Magnetic Resonance Imaging

Data was acquired on a 4.7 T Bruker PharmaScan 47/16 (Bruker BioSpin GmbH), equipped with a volume resonator operating in quadrature mode for excitation and a four-element phased array surface coil for signal reception. During MRI acquisition, mice were kept under isoflurane anesthesia 1.5% in a 4:1 air/oxygen mixture. Body temperature was monitored with a rectal temperature probe (MLT415, ADInstruments) and kept at 36 ± 0.5°C using a warm water circuit integrated into the animal support (Bruker BioSpin GmbH). First, one sagittal 2D slice was acquired with a true fast imaging with steady state precession (TrueFISP) sequence to reproducibly position the image slices for the *T*_2_ map between animals and different days of assessment. The TrueFISP sequence was applied with the following parameters: echo time (TE) = 3 ms, repetition time (TR) = 6 ms, 100 averages, slice thickness = 1 mm, field of view (FOV) = 2.56 cm × 1.28 cm, and matrix size = 256 mm × 128 mm, giving an in-plane resolution (IR) = 100 μm × 100 μm. For time-of-flight MR angiography a 3D FLASH sequence with the parameters TE = 3 ms, TR = 12 ms, α = 20°, 3 averages, FOV = 20 mm × 20 mm × 20 mm, and a matrix size = 256 mm × 256 mm × 256 mm, resulting in an IR = 78 μm^3^ were acquired. To assess the *T*_2_ relaxation time of muscle tissue, a 2D Carr-Purcell-Meiboom-Gill multi-slice-multi-echo sequence was performed with 14 echoes, TE1 = 12 ms, an inter-echo time = 12 ms, TR = 2783 ms, averages = 4, FOV = 20 mm × 20 mm, and matrix size = 100 mm × 100 mm, giving an IR = 200 μm × 200 μm.

### MSOT Image Reconstruction and Spectral Unmixing

**Figure 1:**
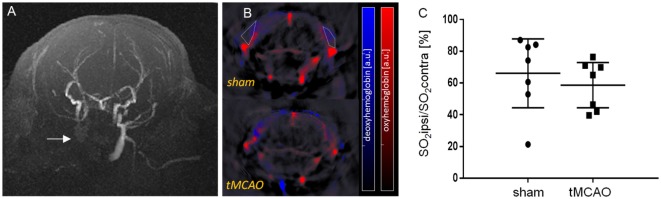
***In vivo* assessment of temporal muscle oxygenation with multi-spectral optoacoustic imaging**. **(A)** Axial maximum-intensity projection of a TOF-angiogram 24 h after transient MCAO (tMCAO). Arrow points to non-perfused common carotid artery and external carotid artery. **(B)** Axial cross-sectional multi-spectral optoacoustic tomography images of mice after 30 min of sham surgery and tMCAO (approximately at Bregma −1.7 ± 0.3 mm) with unmixed signal from oxygenated (red) and deoxygenated (blue) hemoglobin, overlaid on the single wavelength image (900 nm). Examples of region of interest are shown (white dotted line). **(C)** Ratio of sO_2_ between the ischemic ipsi- and contralateral temporal muscles. Mean with 95% confidence intervals, sham: *n* = 7, tMCAO: *n* = 7.

Images were reconstructed using a backprojection algorithm with a non-negativity constraint imposed during inversion (7). Linear unmixing was applied to resolve signals form deoxygenated and oxygenated hemoglobin (8). For each pixel in the image, the method fits the total measured optoacoustic spectrum to the known absorption spectra of oxy- and deoxy-hemoglobin. On axial cross sections of the mouse head regions of interest were drawn over the left ischemic (ipsi) and right (contra) temporal muscle regions (Figure 1). Oxygen saturation of each region was calculated according to the following formula:

(1) $${\text{sO}}_{\text{2}}={\text{HbO}}_{\text{2}}/\text{Hb}+{\text{HbO}}_{\text{2}}$$

The ratios of sO_2_ values between the ipsi- and contralateral sides were calculated.

### MRI Data Processing and Analysis

*T*_2_ maps were computed using Paravision software (Bruker) by mono-exponential fitting of the spin-echo magnitude signal decay, *S*,

(2) $$S=S_{0}\;\exp\left(-\frac{\text{TE}}{T_{2}}\right)$$

where both *S*_0_, the signal at TE = 0, and *T*_2_, the irreversible relaxation time, are fitted.

Regions-of-interest were drawn on *T*_2_ maps around the temporal muscle identified on the ipsi- and contralateral side of the mouse head on four consecutive images.

### Histology

Animals were put under deep anesthesia by intraperitoneal injection of ketamine/xylazine/acepromazine maleate (100/20/3 mg/kg body weight) and transcardially perfused with 20 ml PBS, followed by 1% paraformaldehyde [(PFA); pH 7.4] in PBS. Whole heads were removed, skinned, and fixed in 4% PFA for approximately 48 h. After decalcification in EDTA–citric acid buffer (pH 7.5, BioCYC GmbH & Co KG, Potsdam, Germany), coronal section were applied to prepare two slices of the brain, which were then routinely paraffin wax embedded. Consecutive sections (3–5 μm) were prepared and stained with hematoxylin eosin.

### Statistical Analysis

Comparisons between groups were made by a Mann–Whitney rank sum test and Student’s *t*-test (SigmaPlot 12.5). A *p*-value < 0.05 was considered significant.

## Results

All raw data are made available in a data repository (9).

### Arrest of Blood Flow in ECA Territory Caused Reduced Tissue Oxygenation in Temporal Muscle

Ligation of the CCA and ECA led to an arrest of blood flow in the corresponding vessel (Figure 1A) and reduced tissue oxygenation (Figures 1B,C). Quantification of tissue oxygenation in the temporal muscle showed reduced sO_2_ values in 7 out of 10 sham and 7 out of 9 tMCAO animals compared to the contralateral side (Figure 1C, mean ± confidence intervals, sO_2_ sham 66 ± 22% and tMCAO 59 ± 14% vs. 100%, *p* < 0.05, excluding non-responders).

### Tissue Damage in the ECA Territory

**Figure 2:**
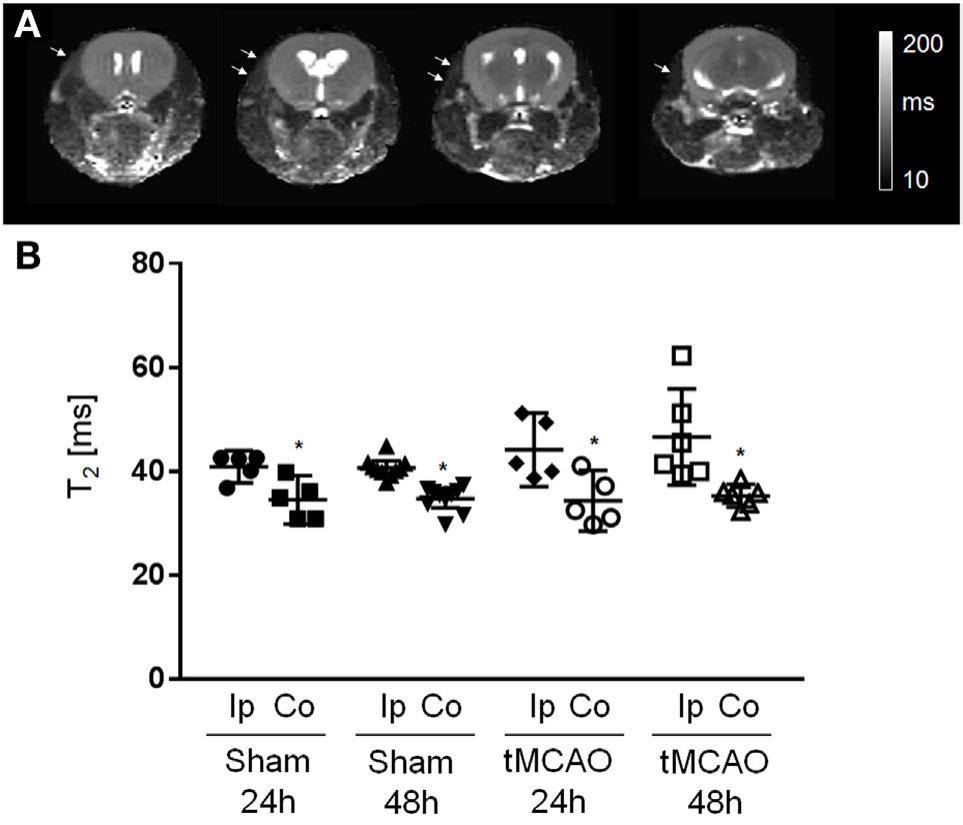
**Assessment of temporal muscle damage with magnetic resonance images**. **(A)** *T*_2_ maps of a mouse head acquired in axial orientation at 48 h after sham surgery. White arrows point to areas of increased *T*_2_ values. **(B)** Regions of interest analysis of *T*_2_ values measured at ipsilateral (Ip) and contralateral (Co) side. Mean with 95% confidence intervals; sham 24 h: *n* = 5, sham 48 h: *n* = 10; transient MCAO (tMCAO) 24 h: *n* = 5, tMCAO 48 h: *n* = 6; *p < 0.05 vs. contralateral.

We inspected parametric *T*_2_ maps of tMCAO and sham-operated animals at 24 and 48 h after surgery (Figure 2A). At the final time point, 10 out of 11 sham and 6 out of 7 tMCAO animals showed increased *T*_2_ relaxation times in the left temporal muscle. Statistical analysis (excluding mice with no differences in *T*_2_ values) showed significantly higher *T*_2_ values between the ipsilateral and contralateral temporal muscle, both at 24 and 48 h after surgery (Figure 2B, mean ± confidence intervals, *T*_2_ sham 40.9 ± 3.1 ms vs. 34.5 ± 4.7 ms and 40.7 ± 1.3 ms vs. 34.7 ± 1.7 ms; tMCAO 44.2 ± 7.1 ms vs. 34.3 ± 5.8 ms and 46.6 ± 9.3 ms vs. 35.3 ± 1.2 ms, at 24 h and 48 h after surgery, respectively, *p* < 0.05), with no differences between sham surgery and tMCAO.

**Figure 3:**
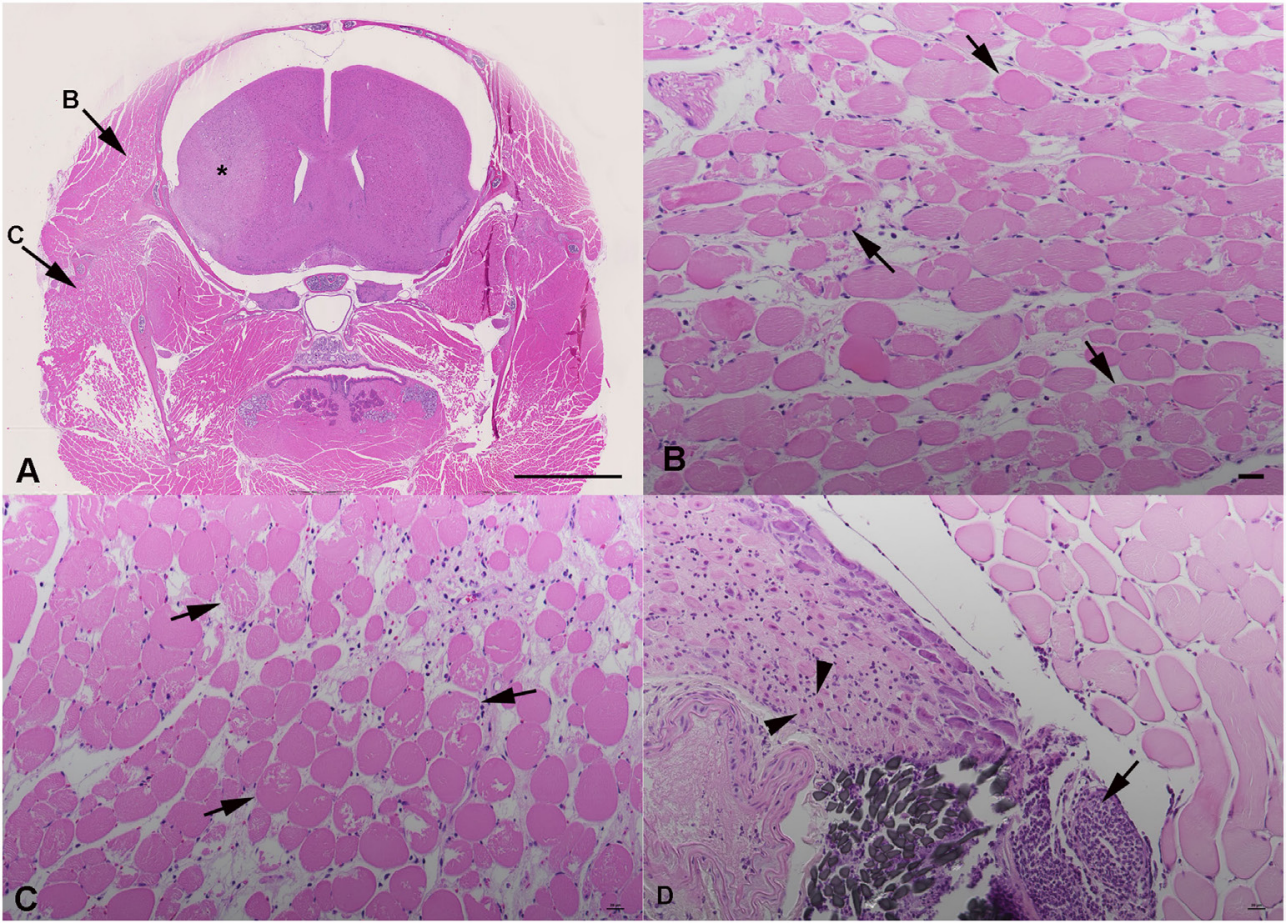
**Histological findings in transient MCAO mouse, 24 h after reperfusion**. Hematoxylin eosin stains. Coronal section of the head at Bregma 1.1 mm **(A–C)**. **(A)** Overview, indicating the cerebral lesion (*), the affected temporal muscle **(B)** and masseter muscle **(C)**. Scale bar = 2.5 mm. **(B)** Ipsilateral temporal muscle with myofibres undergoing degeneration (arrows) and mild interstitial edema. **(C)** Ipsilateral masseter muscle with myofibre degeneration (arrows). There is moderate interstitial edema with several neutrophils in interstitial capillaries. **(D)** Ligature (gray suture material) with intense focal neutrophil infiltration (arrow) extending into the adjacent cervical ganglion. The ganglion exhibits neuronal necrosis (arrowheads) and a mild neutrophil infiltration. **(B–D)** Scale bar = 20 μm.

The histological examination of cross sections prepared from the whole head of tMCAO mice at 24 h after reperfusion (Figures 3A–D) confirmed the presence of degenerated myofibers in the ipsilateral temporal muscle, though of a variable degree (Figure 3B). This was generally associated with evidence of neutrophil recruitment into the muscle and mild interstitial edema. Rare individual degenerate fibers were also seen in the ipsilateral temporal muscle of sham-operated mice (data not shown). The contralateral muscle was unaltered. However, myofiber degeneration was also observed in other muscles of the ECA territory, in particular the masseter muscle bulks, where it was generally more intense than in the temporal muscle, and associated with neutrophil influx (Figure 3C). In addition, intense focal neutrophil-dominated inflammatory infiltration with surrounding edema was present at the site of ECA ligation (Figure 3D). In some cases, this extended to the cervical ganglion and was even associated with neuronal necrosis and neutrophil infiltration of the ganglion.

## Discussion

We demonstrated that ECA ligation required for tMCAO/sham procedure results in acute reduction of tissue oxygenation and in subsequent tissue damage. Generally, a high variability in read-outs was observed between animals. This might be likely due to differences in vascular architecture of individual animals (10). Animals with marked collateral blood supply to the ECA territory are probably less susceptible to ischemia after ECA ligation, whereas littermates with poorer collateralization might tend to develop ischemic damage after the experimental procedure. Given the differences in vascular architecture between different mouse strains (10), the effect of ECA ligation may differ in strains other than C57BL/6.

Skeletal muscle is more tolerant to ischemia than the brain and requires several hours of ischemia before damage manifests (11). In the MCAO model, the ECA ligation is not reversed and we have shown that this leads to increased *T*_2_ values. Histological examination revealed myofiber degeneration, edema, and neutrophil influx in areas of increased *T*_2_. In addition to the ischemic muscle damage, ECA ligation induced local inflammatory processes in the vessel wall and surrounding tissues, where the inflammatory processes can even involve the cervical ganglion.

In the current study, we have not investigated functional consequences of ECA ligation. However, it was previously reported that tMCAO leads to weight loss, ischemia-induced muscle wasting, and reduced food intake, which is correlated with lesion volume (12). Based on the results of the present study, one could speculate that the tMCAO and even the surgery alone can be responsible for some of the decreased food intake, both due to muscle degeneration in the ECA territory and due to surgery-associated local inflammatory processes that are associated with pain and/or affect the sympathetic nervous system. Extensive muscle damage can lead to impaired function of muscles involved in mastication and swallowing and myalgia. Studies have previously described the impairment of motor function in the intraluminal tMCAO model in rats (2, 3). Moreover, ischemia of the skeletal muscles leads to a systemic inflammatory response (13), which might affect the dynamics and distribution of immune cells in the tMCAO model. Indeed, we reported an increase in the proportion of circulating neutrophils in the blood of sham-operated animals similar to tMCAO mice (4). Also, muscle ischemia leads to factor XII-driven contact-induced activation of the coagulation system and can induce coagulopathy (14). Though the execution and duration of the surgery might affect these described parameters, also the type of anesthesia and analgesia, the effects of ECA tissue damage on the read-outs need to be considered (15, 16). A possible solution to prevent muscle damage in the ECA territory is to reperfuse the CCA and the ECA also after retraction of the filament. This would require closing the incision in the CCA and might thus be technically very challenging.

In summary, ECA ligation leads to variable tissue damage. Its confounding impact on study parameters in experimental stroke studies needs to be further investigated.

## Author Contributions

MV, AK, and JK conceived and designed the experiments. MV, RN, and AK performed the experiments. MV, RN, AK, and JK analyzed the data. MV, RN, AK, MR, and JK interpreted results. JK wrote the paper. All coauthors made critical revisions to the manuscript.

## Conflict of Interest Statement

The authors declare that the research was conducted in the absence of any commercial or financial relationships that could be construed as a potential conflict of interest.
